# *Otodectes cynotis* (Acari: Psoroptidae) infestations in Southern pudus (*Pudu puda*): *In situ* and *ex situ* data of an unexpected host-parasite record

**DOI:** 10.1016/j.ijppaw.2025.101043

**Published:** 2025-01-28

**Authors:** Caroline Wilhelm, Edwin Kniha, Pamela Muñoz, Ángelo Espinoza, Laura Platner, Saskia Dreyer, Lisa Grund, Zoë Tess Lara Lindhorst, Ulrich Gärtner, Julia Walochnik, Anja Taubert, Dominik Fischer, Stephan Hering-Hagenbeck, Carlos Hermosilla, David Ebmer

**Affiliations:** a Vienna Zoo, Vienna, Austria; b Institute of Parasitology, Biomedical Research Center Seltersberg, Justus Liebig University Giessen, Giessen, Germany; c Institute of Specific Prophylaxis and Tropical Medicine, Center for Pathophysiology, Infectiology and Immunology, Medical University of Vienna, Vienna, Austria; d Laboratorio de Parasitologia Veterinaria, Instituto de Patología Animal, Facultad de Cs. Veterinarias, Universidad Austral de Chile, Valdivia, Chile; e Centro de Rehabilitación de Fauna Silvestre (CEREFAS), Facultad de Ciencias Veterinarias, Universidad Austral de Chile, Valdivia, Chile; f Der Grüne Zoo Wuppertal, Wuppertal, Germany; g ZOOM Erlebniswelt, Gelsenkirchen, Germany; h Institute of Anatomy and Cell Biology, Justus Liebig University Giessen, Giessen, Germany

**Keywords:** Zoological gardens, Otodectic mange, Hair loss, Skin disease, Ectoparasites

## Abstract

*Otodectes cynotis* (Acari: Psoroptidae) constitutes an obligate, non-burrowing ectoparasite and causes otodectic mange primarily in domestic and wild carnivores. Only few studies have described this parasite in herbivore hosts so far. In the current study, we report *O. cynotis* infestations in Southern pudus (*Pudu puda*), categorized in the IUCN red list as near threatened. *Otodectes cynotis* was detected in free-ranging animals in Chile (*in situ*), as well as in zoo-housed pudus at the Zoo Wuppertal, Germany (*ex situ*). During clinical work, two free-ranging pudus temporarily rehabilitated at the Centro de Rehabilitación de Fauna Silvestre (CEREFAS), Valdivia, Chile, were observed with low to moderate yellowish-brown secretions and encrustations inside the pinna and external auditory canal accompanied by an inflammatory *Otitis externa*. Analysis via light microscopy exhibited the presence of mange mites, which were identified as *O. cynotis* via morphological characteristics and molecular analysis. At the Zoo Wuppertal, ear mites were detected in 15 pudus between 2015 and 2024, however, a definite species identification (*O. cynotis*) was carried out in 4 animals within the current study between 2023 and 2024. Some affected pudus showed bald spots around the ears and the head and exhibited headshaking behavior, whilst others were asymptomatic. In some cases, mites were found as a secondary finding when clinical examination under general anesthesia was performed for other reasons. To the best of our knowledge, this signifies the first report of *O. cynotis* infestations in pudus by combining morphological and molecular identification. We here present clinical *in situ* and *ex situ* data and show that zoological gardens and widlife rehabilitation centers play an important role in research and monitoring of neglected wildlife diseases.

## Highlights

• *Otodectes cynotis*, typically parasitizing carnivores, in an unusual host-parasite relationship.
• First *in situ* detection of *O. cynotis* in Southern pudus in Chile.
• Analysis of *O. cynotis* via light and scanning electron microscopy.
• First cytochrome oxidase 1 (COI) sequences of *O. cynotis* infesting herbivorous hosts.

## Graphical abstract

**Figure:**
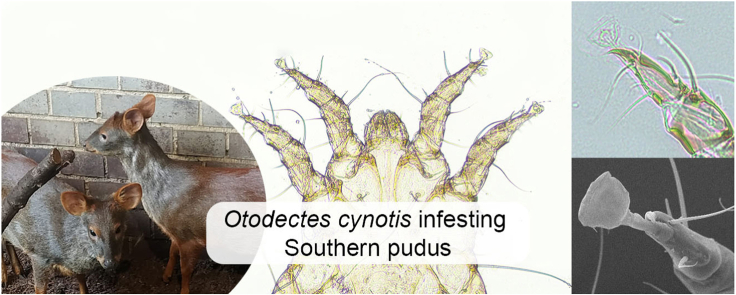

## 1 Introduction

Psoroptic mites of the species *Otodectes cynotis* (Hering, 1838) are obligate, non-burrowing parasites and the causative agents of otodectic mange (Sweatman, 1958). They are primarily associated with domestic and wild carnivores (e.g. dogs, cats, ferrets, foxes and raccoons) worldwide and mainly infest the external acoustic meatus (Mullen and O’Connor, 2019). *Otodectes cynotis* resides through all stages (egg, larva, protonymph, deutonymph and adults) in the ear (Sweatman, 1958), where they cause an *Otitis externa*, characterized by moderate signs like ear itching, inflammation, dark brown waxy aural debris up to excessively dark cerumen (“coffee ground-like”), head shaking and, in some cases, bloody discharge (Orcutt and Tater, 2012; Mullen and O’Connor, 2019). Less commonly, mites may also parasitize other parts of the host's body (Orcutt and Tater, 2012; Mullen and O’Connor, 2019). Severe, untreated otodectosis cases have been described to result in emaciation, self-induced trauma, convulsions, and twitching (Sweatman, 1958; Mullen and O’Connor, 2019). Furthermore, cases of mortality caused by heavy parasite burdens have been reported when parasites were newly introduced into previously unaffected populations, highlighting the critical risks associated with such infestations (Goltsman et al., 1996).

*Otodectes cynotis* mites can be transmitted from host to host by direct contact. Host switching is especially likely to occur between mother and offspring due to close physical contact during the neonatal period (Mullen and O’Connor, 2019). Even though *O. cynotis* constitutes an obligate ectoparasite, mites can survive outside the host for up to twelve days, depending on environmental factors (Otranto et al., 2004). Most studies on *O. cynotis* focus on domestic animals, especially on dogs and cats (Yang and Huang, 2016; Fanelli et al., 2020). In recent years, however, an increasing number of studies described an occurrence of this mite species in wild carnivores (Briceño et al., 2020; Huang-Bastos et al., 2020). Furthermore, *O. cynotis* should be considered as potential zoonotic parasite, since reports in humans exist, but they are very rare (Cakabay et al., 2016).

With a body length of <85 cm and a shoulder height of <50 cm, the Southern pudu (*Pudu puda)* is one of the smallest members of the deer family (Cervidae) (Hershkovitz, 1982). The Southern pudu is native to the South American Andes of Chile and Argentina (Hershkovitz, 1982) and represents one of the least studied mammals of the Chilean forest fauna (Weber and Gonzalez, 2003). According to the International Union for Conservation of Nature (IUCN), the pudu is classified as near threatened (NT) with an estimated population of 10,000 individuals (Silva-Rodríguez et al., 2016). Particularly, anthropogenic factors pose a threat to this small deer species and populations have declined significantly in recent years, mainly due to forest loss and landscape fragmentation (Silva-Rodríguez et al., 2010). Other important causes for the decline are diseases transmitted by pets and farm animals (Jimenez, 2010). Studies also showed that the pudus' habitat is barely represented in the Chilean system of national protected areas (Pavez-Fox and Estay, 2016). In Central Chile, steadily increasing agricultural and forestry land use is associated with landscape fragmentation and hence loss of suitable habitats. In addition, it has been suggested that Chilean pudus adapt their spatio-temporal ecology as a mechanism to reduce predation risk (Zúñiga and Jiménez, 2018). Thus, the pudu is also threatened by predation from feral and domestic dogs as well as poaching (Silva-Rodríguez et al., 2010). However, due to its evasive behavior, this species has hardly been studied in its natural habitat (Pavez-Fox and Estay, 2016).

*Ex situ*, a pudu was first exhibited at the Berlin Zoo in 1896 (Frädrich, 1980). In South America, the first report of pudus in captivity for breeding purposes dates back to 1953 (Housse, 1953). In Chile, the majority of captive pudus are now held in rehabilitation centers (Jimenez, 2010). According to the Species 360 ZIMS (Zoological Information Management Software: https://www.species360.org) database, a total of 65 Southern pudus are currently housed across twenty European zoological institutions (accessed February 12, 2024).

*Otodectes cynotis* has occasionally been detected in unusual hosts, namely in anteaters (Myrmecophagidae*,* Edentata) (Diniz et al., 1995), Patagonian cavies (*Dolichotis patagonum*) (da Cruz et al., 2017), and even Southern pudus (Schmäschke et al., 2005; Siepel et al., 2016). In the current study, we report clinically manifested cases of otodectic mange in free-ranging Southern pudus in Chile (*in situ*), as well as in pudus held at the Zoo Wuppertal (*ex situ*). Furthermore, we present morphological and, for the first time, molecular identification of *O. cynotis* infesting Southern pudus.

## 2 Material & methods

### *2.1* *In situ* sampling and clinical picture

**Fig. 1:**
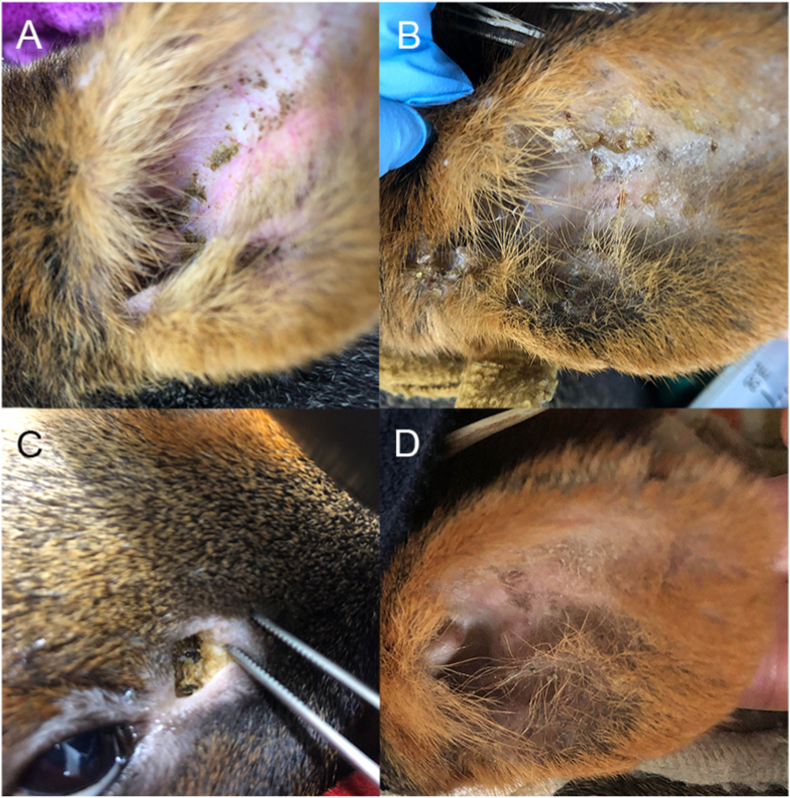
**Clinical presentation of *O******todectes******cynotis* infestation in free-ranging Southern pudus (*Pudu puda*) in Chile:** Left ear pinna with (A) low-grade and (B) moderate waxy brown-yellow ear discharge with encrustations. (C) Periorbital gland with yellow secretion. (D) Left ear pinna four weeks after ivermectin treatment.

During a period of one month (March 21 to April 20, 2023) two wild Southern pudus were sampled for mites at the Centro de Rehabilitación de Fauna Silvestre (CEREFAS) of the Universidad Austral de Chile (UACh) in Valdivia, Chile. Both animals were rescued due to severe hoof burns caused by wildfires in Chile in February of 2023. The sampled animals were a juvenile (< one year old) male and an adult female (> one year old). During routine hoof bandage changes, conducted every two to four days under brief gas anesthesia with isoflurane, the pudus presented with mild to moderate yellowish-brown ceruminous exudate and crusting, accompanied by mild to moderate erythema of the pinnae and external auditory canals (Fig. 1). Crusts were removed from the ears of both pudus and placed on microscope slides. After the initial assessment of the material, during which mites were suspected, the periorbital glands of the animals were subsequently sampled as suggested in the literature (Schmäschke et al., 2005).

### *2.2* *Ex situ* holding, sampling and clinical signs

At the Zoo Wuppertal, several groups of Southern pudus are housed in different enclosures. In the first stage of the study in April 2023, nine animals (three males, six females) were held at three different exhibits. Breeding groups usually consist of one male and one to two females and, after cubbing season in May and June, also their offspring. Offspring are transferred to other enclosures when they reach sexual maturity. Pudu enclosures at the Zoo Wuppertal usually include an indoor and an outdoor area. Outdoor exhibits regularly comprise grass, soil or sand floors and are equipped with bushes and trees. In the indoor enclosure, visible for zoo visitors, big roots, woodpiles and wooden shelters are available to the animals. In one enclosure-complex, pudus are housed with Chacoan mara (*Dolichhotis slinicola*) and Baird's Tapir (*Tapirus bairdii*). The *ex situ* population of *P. puda* is managed in an EEP (EAZA Ex situ Programme) coordinated by the Zoo Wuppertal. In the EEP framework, regular animal transports between different zoos are conducted. During this study, one male pudu (born in May 2022, pudu ID #04) was imported to the zoo in August 2023.

In April 2023, one female Pudu (pudu ID #01) was observed with bald spots on the forehead and an increased licking behavior at the Zoo Wuppertal. Moreover, the animal allowed a Chacoan mara to lick the inside of her pinnae. A general anesthesia (5 mg/kg ketamine, Ketamine 10%, Medistar and 0.09 mg/kg medetomidine, Dorbene vet® 1 mg/ml, Zoetis) was performed for thorough clinical examination. Samples were taken with a cotton swab from both ear canals and periorbital glands. One part of the collected material was placed directly on a microscopic slide while the other part was preserved in 80 % ethanol and sent to the Parasitology Lab of the Vienna Zoo for parasite identification*.* Anesthesia was antagonized with 0.4 mg/kg atipamezole (Alzane® 5 mg/ml, Zoetis) intramuscularly.

Between 2015 and 2024, aural mite infestations had been detected in 15 pudus at the Zoo Wuppertal and clinical signs, which were not present in all cases, included „shaggy fur“, bald spots around the head, especially face and neck, head shaking and licking behavior. Mites were found during clinical examination under general anesthesia as described above. However, prior to the start of this study in 2023, no detailed morphological identifications and species determinations of ear mites were made. During the current study, ear swabs of four individuals were screened in April 2023 and May 2024 at the Parasitology Lab of the Vienna Zoo (Table 1). In affected pudus, the ears were often filled with yellowish, crumbly cerumen and in a few animals, the secretion within the periorbital glands was very firm, dry and appeared stone-like. In May 2024, Chacoan mara and Baird's tapir, inhabitants of the multi-species exhibit, were also tested using ear swabs. Therapy control was mainly evaluated by the monitoring of clinical symptoms by veterinarians and zookeepers on a daily basis. To minimize stress, animals were not regularly handled and tested after treatment.

**Table 1:** Results of the samples screened from pudus held at Zoo Wuppertal.

Pudu ID	Sex	Date of birth	Place of birth	Date of transport to Zoo Wuppertal	*O. cynotis* monitoring
**April 2023**					
#01	female	24/05/2020	France	05/07/2022	**positive**
#02	female	20/05/2022	Wuppertal (offspring)	–	negative
#03	female	17/06/2022	Wuppertal (offspring)	–	negative

**May 2024**					
#04	male	08/05/2022	Germany	22/08/2023	**positive**
#02	female	20/05/2022	Wuppertal (offspring)	–	**positive**
#03	female	17/06/2022	Wuppertal (offspring)	–	**positive**

### 2.3 Antiparasitic treatment and treatment control

Treatment of the wild pudus was performed using 0.2 mg/kg bodyweight (bw) ivermectin (Ivomec® 1 %, Merial) subcutaneously, a therapy chosen due to the widespread use of the drug in wildlife medicine and its status as the standard recommended dose for ruminants (Dieterich and Craigmill, 1990; Panayotova-Pencheva, 2016). The treatment was repeated after 10 days as part of the protocol. *Ex situ*, ivermectin was initially administered subcutaneously at the same dosage (0.2 mg/kg) three times at intervals of 14 days in Zoo Wuppertal. As this regimen resulted in reinfestations, a new treatment protocol was implemented, involving topical ivermectin (0.1 ml ivermectin 1% mixed with 0.1 ml propylene glycol in each ear) and eprinomectin (Eprinex® 5 mg/ml, pour-on, Merial), repeated after 14 days. Eprinomectin had been previously documented as effective in eliminating Sarcoptes mites in cattle (Schoenberg, 1999). Since 2023, eprinomectin was diluted 1:1 with propylene glycol and inserted directly into the ears and periorbital glands. This treatment was also applied to all other animals in the multi-species enclosure. The efficacy of the therapy was mainly checked otoscopically and by examining aural swab samples, both *in-* and *ex situ.*

### 2.4 Morphological examination of mites

First examinations on the presence of parasites were carried out using a light microscope. Aural crusts and dander were placed on a microscope slide and covered with immersion oil and a cover slip. For an extended morphological identification at the Vienna Zoo, 16 mites (ten females, two males and four larvae) from wild pudus and multiple mites from pudus held at the Zoo Wuppertal were isolated out of the danders, embedded in Hoyer's medium and examined using a light microscope (Olympus BX50, Olympus, Tokio, Japan) equipped with a digital camera (Olympus EP50). The specimens were measured, photographed and identified on the basis of established morphological characteristics (Sweatman, 1958; Lohse et al., 2002).

For scanning electron microscopy (SEM), a male *O. cynotis* preserved in 80 % ethanol was used. The specimen was positioned on a circular (10 mm of diameter) glass coverslip (Nunc) which was pre-coated with poly-_L_-lysine (Sigma-Aldrich). Then the specimen was prepared in a standardized procedure as follows: fixation in 2.5 % glutaraldehyde (Merck), post-fixation in 1 % osmium tetroxide (Merck), washing in distilled water, dehydrating, critical point drying by CO_2_ treatment. Afterwards, the sample was sputtered with gold. The mite was examined by using a Philips XL30® (Amsterdam, Netherlands) scanning microscope at the Institute of Anatomy and Cell Biology at Justus Liebig University Giessen, Germany.

### 2.5 Molecular identification and genotyping of *O. cynotis*

Mites were preserved in 70 % ethanol and sent to the Institute for Specific Prophylaxis and Tropical Medicine at the Medical University of Vienna for further molecular analysis. Mites were rinsed twice with 70 % ethanol, cut in half with sterile needles under the stereomicroscope, and individually transferred to 1.5 ml tubes. Afterwards, 180 μL buffer ATL and 20 μL Proteinase K were added and the mixture was incubated at 56 °C while shaking at 500 rpm for 4 h. Then, DNA was then isolated using a QIAamp® DNA Blood & Tissue Kit (Qiagen, Hilden, Germany), following the manufacturer's intructions. Molecular identification (barcoding) was based on amplification of the widely used cytochrome *c* oxidase 1 (COI) gene segment using the primer combination LCO1490/HCO2198 and PCR conditions published previously (Folmer et al., 1994). Genoytping was based on the internal transcribed spacer 2 (ITS2) as proposed by Lohse et al. (2002) using the primer combination RIB-4CB (Briceño et al., 2020) and RIB-31 (Lohse et al., 2002), and a modified PCR protocol (initial denaturartion: 94 °C for 5 min) published by Briceño et al. (2020). PCRs were run on an Eppendorf Mastercycler (Eppendorf AG, Hamburg, Germany) using a 2x EmeraldAmp® GT PCR Master Mix (Takara Bio Europa SAS, Saint-Germain-en-Laye, France) with 3 μL template DNA and sterile H_2_O at a final volume of 25 μL. Purified bands were sent to Microsynth (Microsynth Austria, Vienna, Austria) for Sanger sequencing, obtained from both strands and aligned with Clustal X2.1 (Larkin et al., 2007). Consensus sequences were generated with GenDoc 2.7.0 (Nicholas and Nicholas, 1997). The obtained sequences were uploaded to GenBank (accession numbers COI: PP691139–PP691144, ITS2: PQ870433–PQ870438) and compared to available sequences using the Basic Local Alignment Search Tool (BLAST) (https://blast.ncbi.nlm.nih.gov/Blast.cgi). For genotyping, available ITS2 reference sequences were downloaded from GenBank, aligned, and genotypes were identified with DnaSP v.5 (Librado and Rozas, 2009). MEGAX (Kumar et al., 2018) was used to illustrate variable sites in the alignment.

## 3 Results

### 3.1 Morphological identification

**Fig. 2:**
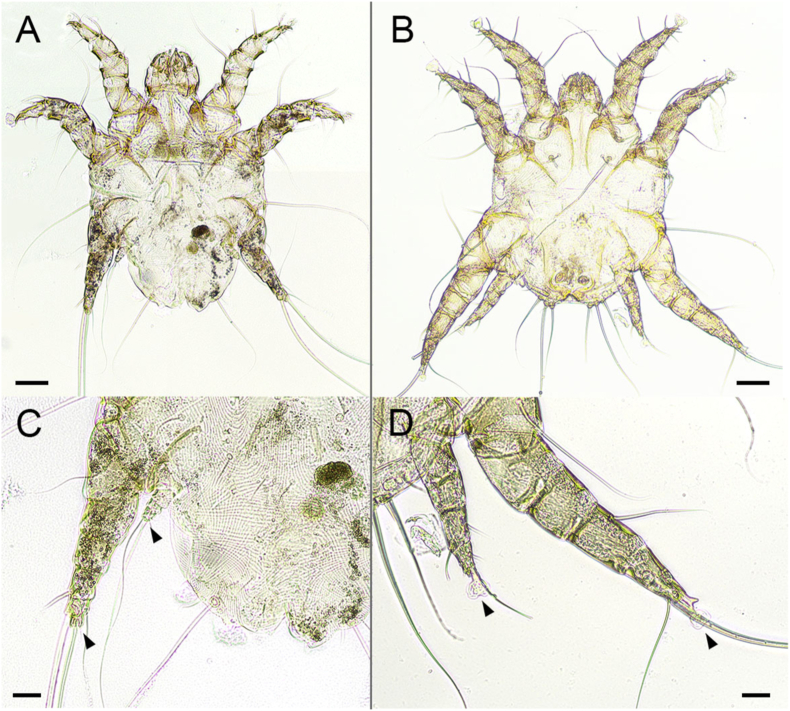
**Light microscopic images of *Otodectes cynotis*.** (A) Overview of female (scale bar: 50 μm) and (B) male mite (scale bar: 50 μm). (C) Posterior part of female mite, showing the absence of pulvilli (arrows) on legs III and IV (scale bar: 20 μm). (D) Presence of pulvilli (arrows) on legs III and IV of male specimen (scale bar: 20 μm).

**Fig. 3:**
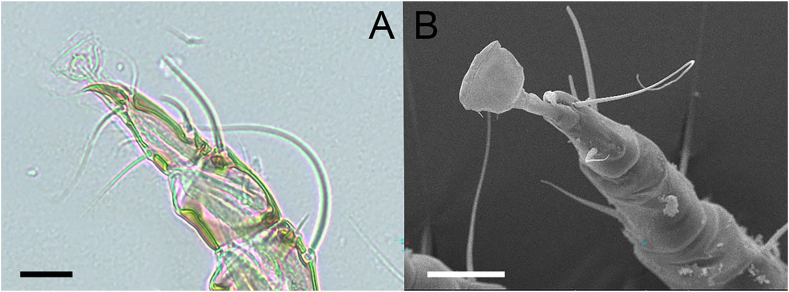
(A) Light microscopic (scale bar: 10 μm) and (B) scanning electron microscopic (SEM) image (scale bar: 20 μm) of the stout inarticulated pulvillus on *Otodectes cynotis* leg II.

Assessing the body shape, length and width, shape of legs and pulvilli, all mites were identified as *Otodectes cynotis* (Acari: Psoroptidae). Male mites presented pulvilli on unspanned pedicles on all legs, while female mites showed the absence of pulvilli on legs III and IV (Fig. 2, Fig. 3).

### 3.2 Molecular identification and haplotyping

Altogether, six COI sequences of *O. cynotis* were obtained with a final length of 639 bp (without primers), of which one originated from the captive pudu at the Zoo Wuppertal and five from rehabilitated Chilean pudus. No nucleotide differences were detected within those sequences. Blasting identity against available sequences in GenBank resulted in 98.75 % (KP676682, KP676683) to 99.84 % (KP676688). All reference COI sequences available in GenBank originated from *O. cynotis* infesting either dogs or cats in China, except for two sequences originating from a cat in India (OQ683857) and a cat in Michigan, USA (KF891933).

Using all available ITS2 sequences of *O. cynotis* from GenBank, 17 sequences (11 from Genbank, 6 from this study) with a length from 321 to 324 bp consisting of 13 genotypes (genotype diversity: 0.9632) were included in the analysis (Suppl. file 1). Altogether, 17 variable sites were detected including three positions in the alignment with gaps. Six obtained sequences (in this study) with a length of 322–323 bp were divided in five genotypes. The sequence originating from the captive pudu at the Zoo Wuppertal (PQ870433) was 100% identical to genotype I (AF367699) identified by Lohse et al. (2002) from *O. cynotis* of dogs (*Canis lupus familiaris*), cats (*Felis catus*), arctic foxes (*Vulpes lagopus*), and ferrets (*Mustela furo*). Sequences from free-ranging *Pudu puda* from Chile comprised four haplotypes, one (PQ870437) being 100% identical to genotype II (AF367700) identified by Lohse et al. (2002) from *O. cynotis* of cats in Chile as well as of dogs in China (KP676677) (Juan et al., 2015). The other three genotypes (genotype VI–VII) were newly identified, with genotype VII comprising two sequences (PQ870436 and PQ870438). Additional genotypes (genotype IX–XI) comprised sequences from cats (KP676675) and dogs (KP676676, KP676677) from China. Two more tentative genotypes (genotype XII, XIII) comprising two sequences of *O. cynotis* infesting Fuegian foxes (*Pseudalopex culpaeus*) in Chile were already identified by Briceño et al. (2020), however, both containing wobble bases (unknown nucelotide) and thus might display genotype I or new genotypes, depending on the missing nucleotide.

### 3.3 Clinical follow-up and treatment success

Both pudus housed at the CEREFAS were diagnosed with *O. cynotis* infestations. Following subcutaneous ivermectin injections, the mild to moderate yellowish-brown ceruminous exudate and crusting within the pinnae and external auditory canals, accompanied by mild to moderate erythema associated with otitis externa, progressively decreased within two weeks (Fig. 1). While the juvenile pudu exhibited clean periorbital glands, a yellowish, tallow-like secretion was observed in the glands of the adult animal. No *O. cynotis* mites were detected in the material sampled from the periorbital glands of either animal. Additionally, no pruritus or other clinical signs of discomfort related to mite infestation were observed in either pudu. During follow-up monitoring, conducted every second anesthesia procedure for hoof bandage changes, whole crusts and swab samples from the external auditory canals were collected and microscopically examined for mite counts. A significant reduction in mite burden was observed, corresponding with a marked improvement in clinical signs. By the fourth week, the encrustations of the external ears had largely resolved, the yellowish-brown ceruminous exudate was absent, and the ears exhibited only mild residual erythema without any evidence of mites on microscopic examination.

At the Zoo Wuppertal, *O. cynotis* was detected in ear swabs of four pudus (Table 1). Two of these positive animals (pudu #01 and #04) were born in different zoos and were transported to the Zoo Wuppertal in 2022 and 2023, the other two (pudu #02 and #03) were born at the zoo. Bald spots on the forehead and increased licking behavior in one female (#01) improved following antiparasitic treatment with ivermectin and eprinomectin. This treatment seemed sufficient for mite removal in all cases regarding clinical symptoms. Examinations of the Chacoan maras and Baird's tapir in the same enclosure in May 2024 denied *O. cynotis* infestations.

## 4 Discussion

In recent decades, wildlife diseases and the health status of animal populations gained importance as part of the One Health concept. Thereby, rehabilitation centers and zoos have a crucial role in research and surveillance of wildlife diseases (Yabsley, 2019). Sampling wild animals is often challenging, which is why animals that can be accustomed to regular handling in zoos or that are temporarily kept in sanctuaries offer opportunities for pathogen monitoring and research in line with animal welfare standards. In this study, we detected infestations with *Otodectes cynotis* in both zoo-held pudus in Germany and, for the first time, in wild animals in a Chilean rehabilitation facility. A thorough clinical examination and the detection of ear mites in pudus requires at least the manual restraint of the animal, but general anesthesia is advisable to enable safe otoscopy, swab sampling and treatment. Due to the need of treatment of burn wounds in rescued animals at CEREFAS, we could sample them without added stress. At the Zoo Wuppertal, ear mites were mainly found when animals were anesthetized for other reasons, such as clinical examination for transportation. Since handling times in general should be reduced to a minimum, parasite sampling should be integrated in the standard procedure of regular health checkups for wildlife in captivity to gain more insights into treatment success and infestation pattern. Minimally invasive sampling methods for the diagnosis and monitoring of wild animal diseases have become increasingly important in recent years (Schilling et al., 2022). Their intention is to minimize the stress of the sampled wild animals and to further increase animal welfare.

Beside *O. cynotis*’ typical host range consisting of different domestic and wild carnivores, case reports in other hosts have previously been published (Diniz et al., 1995; Schmäschke et al., 2005; Siepel et al., 2016; da Cruz et al., 2017). In the case of pudus, *O. cynotis* was already diagnosed in European zoos: Schmäschke et al. (2005) published the first evidence of *O. cynotis* in six pudus living at the Halle Zoo, followed by a further record from the Rotterdam Zoo (Siepel et al., 2016). To the best of our knowledge, in herbivore hosts, so far, the diagnosis of *O. cynotis* has exclusively been conducted by morphological parasite identification. This study represents the first molecular identification of *O. cynotis* in pudus, as well as the first detection of this ectoparasite in free-ranging pudus from Southern Chile, providing tangible evidence of the pudu as a host for *O. cynotis*. Despite not being regularly applied for Acari, cytochrome oxidase 1 displays a reliable marker gene for species discrimination of *O. cynotis*, especially since no sequences are available in GenBank for the more commonly applied 16S rRNA gene, so far (Black and Piesman, 1994).

The clinical signs of otodectosis observed in pudus closely align with those documented in literature for carnivores (Sweatman, 1958; Mullen and O’Connor, 2019). However, unlike the characteristic dark, coffee grounds-like cerumen commonly seen in ear mange of dogs and cats, the pudus displayed a waxy brown-yellow ear discharge with encrustations. Despite accompanying inflammatory otitis, no signs of pruritus were evident in the wild pudus. However, it is important to approach this statement with caution. The inherent shy nature of the animals likely led to heightened stress at the rehabilitation center. Due to the need for rest, they were not continuously monitored and behaviors indicative of pruritus may have been overlooked. Additionally, the stressful environment may have suppressed the animals' natural behaviors. In the Zoo Wuppertal, licking behavior as a potential indicator for itching could be observed in some animals. They also exhibited broken hair in the area around the mouth, where they could reach to lick. Animals were checked daily and were calm in their indoor hiding places. Most animals did not show obvious clinical signs. In European zoos, mites have previously been found in the ear canal and the periorbital glands, as described by Schmäschke et al. (2005). The current wild animals sampled in the rehabilitation center did not show infestations in the periorbital glands. Infestations in various body parts, like the perineum or tail tip, have been documented at rare instances across different species (Orcutt and Tater, 2012; Mullen and O’Connor, 2019). The infestation of periorbital glands in pudus might represent a rare but effective colonization site, since they provide a favorable environment and a protected niche for ear mites in chronical cases. Given the significant size of these glands in pudus, they should generally be considered in treatment protocols.

A 0.2 mg/kg bw s.c. ivermectin application, administered in two cycles with a 10-day interval, significantly alleviated symptoms and eventually eliminated *O. cynotis* in wild pudus at the rehabilitation center. However, the successful elimination of *O. cynotis* mites from pudus at CEREFAS should be approached with caution due to experiences on recurrent and persistent infestations at the Zoo Wuppertal. The short duration of the pudus' stay at the rehabilitation center limits the monitoring of long-term therapy outcomes. In the Zoo Wuppertal, however, ivermectin injections alone were ineffective. Recurring mite infestations in pudus led to adaptations of the treatment protocol. Therefore, initial topical ivermectin was followed by topical eprinomectin application. The latter has been used to rehabilitate cattle herds from mange (Schoenberg, 1999). In mixed species enclosures, the other animals (Chacoan maras and Baird's tapir) were treated with eprinomectin as well, to prevent them from acting as a mite reservoir. Furthermore, eprinomectin was applied directly into the ears and the periorbital glands. However, it should be noted, that therapy monitoring was mainly done via observations of clinical signs, and exact evaluations of the decrease in mite burden were not possible due to animal welfare aspects. Thus, mites potentially could have survived in the pudu groups at the Zoo Wuppertal in undiagnosed reservoir carriers or in the environment. Thereby, monitoring of clinical signs via zookeepers and veterinarians is essential to recognize infestations. Further, regular check-ups of the current infestation status during other medical interventions should be performed to gain a better insight into this parasitosis. Interestingly, two of the pudus that tested positive, had transport histories and were imported from a zoo in France in July 2022 (pudu #01, *O. cynotis*-positive in April 2023) and from a German zoo in August 2023 (pudu #04, *O. cynotis-*positive in May 2024). Transmission might have happened after arrival at the Zoo Wuppertal, however, an occult introduction of parasites via asymptomatic animals does also represent a realistic scenario. However, more studies are required to study actual occurrence and prevalence of otodectic mange in Southern pudus held at European zoos.

Ivermectin is a well-documented broad-spectrum antiparasitic widely used in zoo and wildlife medicine (Panayotova-Pencheva, 2016), but to our knowledge there exist no studies on its application in pudus. In this study, the choice of application is based on the standard dosage for ruminants, as well as successful experiences with related species, such as reindeer (*Rangifer tarandus*), in which ivermectin was safely used at that standard dosage of 0.2 mg/kg bw (Dieterich and Craigmill, 1990). While ivermectin is effective, its frequent use poses risks, including development of parasite resistance as seen in *Haemonchus contortus* (Cazajous et al., 2018; Alkadir et al., 2023) and environmental contamination. It is primarily excreted in feces, where it remains biologically active for up to a month, potentially harming soil organisms and aquatic ecosystems (Wells and Collins, 2022; Adeleye et al., 2023). The prolonged environmental persistence of ivermectin and the associated risk of reinfestation necessitate cautious use in wild pudus. Animals in rehabilitation facilities for extended periods can be treated effectively under supervised conditions. However, in cases of short stays or opportunistic captures treatment with ivermectin should be carefully evaluated. Furthermore young animals are described to react more sensitive to ivermectin due to an underdeveloped blood-brain barrier (Button et al., 1988). It should therefore be considered to adjust dosages in very young pudus with underdeveloped blood-brain barrier to minimize adverse risks. For pregnant animals, ivermectin is considered safe based on studies in other species, including cattle, where it has been shown to be neither embryotoxic nor teratogenic (Campbell and Benz, 1984). It is also deemed safe for lactating animals, as only a small fraction (5%) passes into the milk (Toutain et al., 1988). Selamectin might be a promising alternative treatment for pudus in the future. It has shown success in treating *O. cynotis* in Patagonian maras with a single application (da Cruz et al., 2017). Selamectin is considered less toxic to the environment than ivermectin, as it is metabolized before excretion and has a shorter half-life (Wells and Collins, 2022). It is also less harmful to young animals (Bishop et al., 2000), though further research is necessary to confirm its effectiveness and safety in pudus.

The treatment of *O. cynotis* with doramectin, as reported in the initial documentation of *O. cynotis* infestations in southern pudus by Schmäschke et al. (2005), did not prove to be sustainably effective. Though this may have resulted from reinfestations due to environmental conditions rather than drug inefficacy. Shared bedding, feeding sites, and retreats in enclosures significantly increase reinfestation risks (Schmäschke et al., 2005). Psoroptic mites such as *O. cynotis* can survive for up to 12 days in favorable environmental conditions, spreading through direct animal contact or contaminated surfaces and fomites like transport boxes and cages (Otranto et al., 2004). Effective control measures could include regular cleaning and disinfection of enclosures and isolating contaminated areas for at least 12 days to ensure mite die-off. Contact with potential reservoir hosts, such as stray cats, must also be avoided (Otranto et al., 2004).

Regarding transmission, *O. cynotis* might have been introduced to the German pudu population with the initial wild-caught pudus some decades ago, which is corroborated by the identification of genotype I initially described from various hosts in Chile (Lohse et al., 2002). Eventually, clinical infestations persisted subclinically, and even after treatments, minor mite populations might have remained underdiagnosed. In their natural habitat, the Andean regions of Chile and Argentina (Hershkovitz, 1982), the diminutive Southern pudu faces a growing threat of habitat destruction and displacement driven by human activities (Silva-Rodríguez et al., 2010). These anthropogenic pressures lead to an increasing overlap of pudu habitats and areas of domesticated pets, stray dogs and cats. In Chile, the estimated population of dogs roaming freely or as strays exceeds 3.5 million (Garde et al., 2022), while the number of stray cats remains unquantified. Considering the high density of potentially infected carnivores, inter-species as well as intra-species transmission of *O. cynotis* may represent an important epidemiological factor, particularly given the mites' ability to persist in the environment for extended periods of time (Otranto et al., 2004). Cases of otodectosis have already been reported in Chilean wild foxes (*Pseudalopex* spp., Briceño et al., 2020). Moreover, the possible transmission by stray cats to other vulnerable species like the Patagonian cavy may occur as well (da Cruz et al., 2017). In Europe, *O. cynotis* has also been documented to infest wild carnivores like the red fox *Vulpes vulpes* (Perrucci et al., 2016). Transmission of *O. cynotis* from these carnivores to the pudus residing in the zoo therefore remains another remote possibility, since urban wildlife represents a possible infestation risk for zoo animal taxa (Keiser et al., 2023). Considering that *O. cynotis* genotype I was identified from specimens infesting various domestic and wild animals, such as cats, dogs, ferrets or arctic foxes (Lohse et al., 2002), a broad host range supporting cross-species transmission events is evident. However, ITS2 variability was neither associated with the host species nor geographic origin, which corroborates findings from Lohse et al. (2002). Thus, ITS2 serves as a valuable marker gene on the species level for psoroptid mites, but may be coupled with the analysis of other, currently unavailable, gene sequences (e.g. 18S rDNA, 28S rDNA). Clearly, further research is required to gain insight into the exact transmission pathways of *O. cynotis*. This knowledge would be crucial for evaluating strategies to manage diseases in large populations of stray animals and their interactions with elusive species like the pudus. Past examples, such as the Mednyi Arctic fox (*Alopex lagopus semenovi*), illustrate how anthropogenic activities can introduce parasites that adapt to novel hosts, leading to devastating population declines (Goltsman et al., 1996). These cases underscore the importance of proactive conservation measures and a deeper understanding of parasite dynamics across species boundaries to mitigate similar risks in vulnerable populations.

## 5 Conclusion

Despite being classified as near threatened (Silva-Rodríguez et al., 2016), the Southern pudu continues to face numerous threats in its natural habitat. With our reports on *in situ* and *ex situ O. cynotis* cases, we here call for more research especially on inter- and intraspecies transmission patterns. Comprehensive research efforts are essential to explore the intricate pathways of otodectosis transmission from domestic animals to wildlife and *vice versa.*

## CRediT authorship contribution statement

**Caroline Wilhelm:** Writing – review & editing, Writing – original draft, Methodology, Investigation, Formal analysis. **Edwin Kniha:** Writing – review & editing, Methodology, Investigation. **Pamela Muñoz:** Writing – review & editing, Resources. **Ángelo Espinoza:** Writing – review & editing, Resources, Investigation. **Laura Platner:** Writing – review & editing, Resources, Investigation. **Saskia Dreyer:** Writing – review & editing, Resources, Investigation. **Lisa Grund:** Writing – review & editing, Resources, Investigation. **Zoë Tess Lara Lindhorst:** Writing – review & editing, Investigation. **Ulrich Gärtner:** Writing – review & editing, Investigation. **Julia Walochnik:** Writing – review & editing, Methodology, Investigation. **Anja Taubert:** Writing – review & editing, Funding acquisition. **Dominik Fischer:** Writing – review & editing, Resources, Investigation. **Stephan Hering-Hagenbeck:** Writing – review & editing, Funding acquisition. **Carlos Hermosilla:** Writing – review & editing, Supervision. **David Ebmer:** Writing – review & editing, Writing – original draft, Visualization, Supervision, Project administration, Investigation, Formal analysis, Conceptualization.

## Ethics approval

*In situ* samples of pudus rehablitated at the CEREFAS were taken during routine clinical work and were in accordance to the Chilean Animal Welfare Legislation. *Ex situ* samples of pudus at Zoo Wuppertal were taken in the framework of veterinary treatments due to *Otitis externa*. Sampling procedures as well as preparation of this manuscript was approved by officials of the CEREFAS and the Zoo Wuppertal.

## Competing interests

The authors declare that they have no competing interests.
